# Extra-Uterine Gestation

**Published:** 1898-02-05

**Authors:** 


### EXTRA-UTERINE GESTATION.

In a presidential address delivered before the British Gynæcological Society, Mr. Mayo Robson draws attention to a symptom of ruptured extra-uterine fœtation which, he believes, has not hitherto been described. There is superficial dulness on percussion over the pubes and in either flank, which on deeper percussion gives a resonant note; but what he particularly draws attention to is that, on turning the patient over, this dulness in the flank, then uppermost, persists for some little time, but gradually disappears in a way which he has never found in the case of any other fluid than blood in the peritoneal cavity. It is also interesting to note that in all the acute cases that he has seen there has been no difficulty in making a diagnosis, the symptoms having always been pathognomonic, and it is extremely important to notice that shock and collapse were not allowed to stand in the way of operative treatment. Three of the patients whose cases are related were pulseless at the time of operation, and in all three a pulse had returned at the wrist before the operation was concluded, doubtless owing to transfusion through the peritoneum by the absorption of the saline fluid used in washing out the abdominal cavity to clear it of blood and clot. Even if there be doubt, "it is infinitely better to make a small exploratory incision in the middle line than to stand idly by and allow the patient to die of hæmorrhage, when the simple application of a ligature could save her life." Such an exploratory incision need not be made to enter the peritoneum, as in cases of intraperitoneal hæmorrhage the blood shows through on exposing the parietal peritoneum. Of course, all this has to do with rupture into the peritoneum, not into the broad ligament, which is another affair.

